# Advancing a Systemic Perspective on Multidisciplinary Teams: A Comparative Case Study of Work Organisation in Four Multiple Sclerosis Hospitals

**DOI:** 10.5334/ijic.3745

**Published:** 2018-07-05

**Authors:** Sam Pless, Geert Van Hootegem, Ezra Dessers

**Affiliations:** 1 Centre for Sociological Research, KU Leuven, Parkstraat 45, Leuven 3000, BE; 2 Centre for Sociological Research, HIVA Research Institute for Work and Society, KU Leuven, Parkstraat 45, Leuven 3000, BE

**Keywords:** multidisciplinary teams, work organisation, integrated care, Multiple Sclerosis

## Abstract

**Introduction:**

Many care organisations claim to employ multidisciplinary teams, but the term is used to describe quite different forms of collaboration. A systemic view of the work organisation of care delivery is presented and applied in this article that allows to identify and understand often overlooked yet important differences regarding team composition, working relationships and therapeutic relationships.

**Theory and Methods:**

We used modern socio-technical systems theory to study care delivery for a particular patient population as a system of interrelated activities. The concept of work organisation refers to the way in which the composite task of care delivery is divided into distinct tasks and how these are grouped in either monodisciplinary or multidisciplinary organisational units. The systemic perspective was applied in a comparative case study of four Multiple Sclerosis hospitals.

**Results:**

Among the hospitals, one was characterised by a functional work organisation, with similar tasks grouped in monodisciplinary teams. Cross-disciplinary working and therapeutic relationships were established on an ad hoc basis. The three other hospitals adopted a more process-oriented work organisation (which groups all tasks related to a specific care process within a single, multidisciplinary team). The more process-oriented the work organisation, the more working relationships and therapeutic relationships appeared to be fixed and continuous.

**Conclusion and discussion:**

The systemic view adopted in this study yields a better understanding of multidisciplinary teams through the concept of work organisation. The actual composition of multidisciplinary teams, and the related working and therapeutic relationships will vary depending on the type of underlying work organisation. Further validation of this conclusion will be needed in other settings.

## Introduction

Many care organisations claim to have installed multidisciplinary teams. In a broad sense, multidisciplinary teams consist of care providers from different disciplines that share a common goal, that is, providing care for a particular population of patients. Multidisciplinary teams are associated with better resource utilization, minimization of unnecessary costs, improvements in job performance and quality of working life, and more efficacious outcomes for patients and their families [1,2]. However, very diverse organisational arrangements are suspected to underlie so-called multidisciplinary teams.

A certain degree of opacity seems to be indicative for the integrated care field as a whole. Care integration is the establishment of connectivity, alignment and collaboration within and between the cure and care sector with regard to funding, administration, organisation, service delivery and clinical practice [4]. Through the establishment of enhanced structures and processes for organisational and service integration, care integration practices try to realise integrated care, i.e. to maximize quality of care and quality of life, patient satisfaction and system efficiency for patients [4]. Multidisciplinary teams are an often advanced component of care integration practices.

Nolte [3] states that there is relatively little research on methodological, analytical and conceptual aspects of the use of qualitative approaches in the evaluation of care integration practices. There has been a move, however, towards ‘realistic evaluation’ [5], which involves quasi-experimental methods for evaluating complex interventions that are highly influenced by contextual factors: what works for whom under what circumstances? [3]. In order to install the preconditions for realistic evaluation, however, the need for a more uniform understanding of care integration practices has to be resolved [6]. Several integrated care models, taxonomies and typologies do exist that improve a systematic conceptual understanding of care integration practices [7,8,9]. Yet they often identify components, dimensions and types of care integration, but shed little light neither on the interaction between components, dimensions and types, nor on their inner workings: how do they work in the first place?

Accordingly, we argue that what is needed is not just a systematic but a systemic conceptual understanding of care integration. A systemic perspective takes the relations between all elements within a system into account. This argument is of course not entirely new. See for example Edgren [10], Tsasis et al. [11], Barasa et al. [12] and Tang et al. [13] on care integration from a complex adaptive systems perspective. Here, modern socio-technical systems theory is used because it offers a more elaborate and more abstract analytical toolkit to describe systems as showed in the theoretical part of this paper. General systems theory formalizes the organisation of phenomena into abstract models of elements and their interrelationships. The multidisciplinary CORTEXS (‘Care Organizations: a Re-Thinking Expedition in Search for Sustainability’) research project has tried to develop such a systemic understanding of care integration [14,15]. We envision care systems from the patient standpoint as the whole of care-related activities that try to meet the patient’s care needs. The core of the care system can be seen as the process of care delivery itself, while legal and financial regulations as well as culture, technology and education are preconditions that determine the possibilities for the concrete organisation of the care process.

This article aims firstly to develop a systemic perspective which furthers the understanding of an important component of care integration, namely multidisciplinary teams. From a systemic view, which important differences might be found between supposedly similar multidisciplinary teams? To answer that question, secondly, a comparative case study of four Multiple Sclerosis (MS) hospitals is undertaken. The MS hospitals were chosen because each hospital substantiates that care providers from different disciplines work together as a multidisciplinary team. Data is collected through topic interviews with 31 care providers and analysed using deductive category application. Ultimately, this article aims to advance a better evaluation, comparison and optimisation of care integration sites with regard to multidisciplinary teams specifically.

## Theoretical framework

### Systems theory

Systems theory developed from the 1930s onwards with lectures and publications from Bertalanffy, culminating into the publication of his ‘General system theory: foundations, development, applications’ in 1968 [16]. Cybernetics, chaos theory and complex adaptive systems theory are theories that came forth from systems theory. The common thread in systems theory is that phenomena can be seen as systems that consist of interrelated and interdependent elements. That general notion will be translated here to the context of care systems using modern socio-technical systems theory.

The term ‘socio-technical systems’ was originally coined in the 1950s by Trist and Bamforth [17]. In the 1970s, the modern socio-technical systems theory was developed by de Sitter et al. [18], based on general systems theory, information theory and cybernetics [19,20,21]. The system studied by modern socio-technical systems theory is not an organisation as such, but a primary process [18,22,23,24]. A primary process consists of all necessary tasks to create a product or to deliver a service from start till end, as seen from the perspective of the product or service end user. This is a major difference in comparison to many other approaches which focus on isolated aspects of an organisation (such as HRM, sales, production, finances) separately, without much consideration for the primacy of the primary process from the perspective of the end user.

In the context of this paper, primary processes are care processes and the tasks are all care-related activities. Patients are the end users. For clarity reasons, we will further be using and applying this care-specific terminology in this paper, although all concepts are generically applicable to primary processes outside the healthcare context as well.

### MS care processes

The context of this study is MS care. The set of symptoms and progression of MS varies per individual [25,26]. The impact of MS on an individual’s life course is often marginal when the disease first shows, but evolves to be very invasive in time. The physical and psychological consequences are a heavy burden to bear, especially given the recurrence of relapses. Accordingly, the array of necessary care-related activities throughout the MS care process is broad, varying from simple to complex procedures, and necessitates a high degree of coordination in order to guarantee continuity of care [27,28]. In sum, the MS care process is the whole of care-related activities that try to answer a specific patients’ needs over time.

Furthermore, the care process can be seen a composite task, which can be divided into distinct tasks (i.e. care-related activities) and grouped into organisational units. Each primary process is characterised by such a work organisation. The work organisation is the core of a system in modern socio-technical theory, on which all other organisational aspects rely, such as governance. For more information on these other aspects studied by modern socio-technical theory, see de Sitter et al. [18] and other authors [22,23,24].

Looking at a generalised MS care process in Flanders (Belgium), the composite task of care delivery typically appears to be divided between monodisciplinary care providers such as GPs, home nurses and pharmacists who do not maintain fixed working relationships in what could be called ‘the primary care phase’. The specialized MS hospitals perform care-related activities in what could be called ‘the MS hospital care phase’. The two phases alternate throughout the care process in accordance with the previously mentioned recurrence of relapses.

In this article, we focus on the ‘MS hospital care phase’. From the outside, MS hospitals appear to be multidisciplinary in nature as they assemble care providers from different disciplines, but very diverse work organisations are suspected to underlie the supposedly multidisciplinary teams. The following section delves deeper into the different types of work organisation.

### Functional and process-oriented work organisation

Generally, two opposite work organisation types are juxtaposed: the functional and the process-oriented work organisation [14,18]. The following paragraphs define these two work organisation types and show their implications regarding team composition, working relationships and therapeutic relationships. Team composition is examined because it touches upon the monodisciplinary or multidisciplinary nature of a team, i.e. do care providers present within the team represent one or more different care disciplines? The concept of working relationships further illuminates the nature of the team as the degree is assessed to which care providers from different disciplines are accustomed to working together, which is either on an ad hoc basis or on a fixed basis. The concept of therapeutic relationships refers to the relation between a patient and his care provider [29], which will be categorised as being ad hoc (changing constantly), sequential (changing periodically) or continuous (never changing).

First, a functional work organisation implies that a composite task is divided into distinct tasks, which are grouped into different organisational units per functional area (e.g. medical, paramedical and nursing departments in a hospital). Consequently, care providers from different monodisciplinary organisational units contribute simultaneously to a particular care process yet from within separate units in a care process, thus not being accustomed to working together. Moreover, a functional work organisation also implies that care providers from a particular discipline are regarded as being interchangeable with regard to a specific care process, which leads to ad hoc therapeutic relationships with the patient. A functional work organisation is associated with the so-called functional silos in healthcare, which are related to suboptimal quality of care, inefficient care delivery and low quality of working life for care providers [30,31,32].

Second, a process-oriented work organisation consists of a composite task that is divided into distinct tasks, which are grouped into organisational units per care process (e.g. an integrated stroke unit). Subsequently, care providers from one multidisciplinary organisational unit constantly work together in the context of a particular care process and maintain continuous therapeutic relationships with a particular patient over time. Process-oriented work organisation is related to high quality of care, efficient care delivery and high quality of working life for care providers [33,34,35].

In most cases, work organisations emerge within organisations rather than being designed consciously [36]. Hence, in reality, a work organisation often does not completely accord with the theoretical types of functional or process-oriented work organisation. Partial forms of functional or process-oriented work organisations can be implemented. Nevertheless, the functional and process-oriented work organisation type are useful conceptual points of reference to study work organisations in practice.

## Methodology

### Case Selection

Since the research question can now be refined to ‘how do differences in work organisation relate to team composition and to the continuity of working and therapeutic relationships?’, we choose to perform a comparative case study [37] of four MS hospitals. Comparative case studies are especially suited to answer explanatory research questions because they deal with intrinsic relations that need to be mapped, rather than mere frequencies or incidence.

The case in this study corresponds to the unit of analysis, which was defined as the MS hospital. We focused on MS because the individually varying disease course and symptoms of MS indicate the need for continuous, multidisciplinary care [26]. For the sake of comparability, we studied multidisciplinary teams within the four government-accredited MS hospitals in Flanders (Belgium), which delivered similar services and operate in comparable constitutional contexts [38]. To increase the chance of finding work organisation differences, all four MS hospitals were selected. Despite some differences in financial arrangements, all hospitals offered similar ambulatory and hospitalization services to patients with the same condition. These services specifically were surveyed in order make sure that the primary processes and all necessary tasks were comparable between the hospitals. For the purpose of this study, the primary processes should be similar, while the work organisations differ. Furthermore, differences in legal structure and organisational size can be noted. Two of the hospitals were wholly owned subsidiaries of a general hospital, while the two other hospitals were autonomous. Accordingly organizational size, measured in number of hospital beds, was substantially smaller in the two subsidiaries (i.e. 10 and 20) than in the autonomous hospitals (i.e. 54 and 134). Although large organisations are often associated with functional work organisation, previous research and the organisations involved in this study show that organisational size does not necessarily predetermine the work organisation [39,40]. No further identifying information is given in this regard to assure anonymity of the cases.

### Data Collection

The main source of data for this article was individual interviews with care providers (n = 31) on site, conducted between November 2015 and September 2016. Ethical approval was obtained from the KU Leuven Ethics Committee (nº G- 2015 09 349). Topic interviews were the appropriate means for data collection. To be precise, in-depth information was needed given (1) the fact that care organisations and providers are not necessarily aware of the concept of work organisation, let alone the particular type of work organisation within their organisation and (2) the differences in in vocabulary used to describe the organisation of care delivery between the four MS hospitals. Unawareness regarding the concept of work organisation means that there is no clear-cut information available in company documents such as organisational charts, care guidelines or patient brochures. Additionally, care organisations and providers do not use the same terminology to describe the work organisation. Each organisation and provider has his own language to talk about work organisation. That is why topic interviews were necessary wherein, guided by the available conceptual definitions, the interview responses were interpreted instantaneously to adjust follow-up questions and figure out how the work organisation was actually structured. The processes of data collection and analysis thus intersect, which requires additional methodological precautions as discussed in the section below.

Individualised, open-ended, topical interview questions were necessary to extract the desired information. To ensure consistency in our data collection, a topic guide was used to conduct the interviews, which were prepared using case-related documents, including inspection reports, presentation slides and patient brochures. The common thread throughout the topic guide was the reconstruction of the care process from the patient perspective, which allowed to uncover the nature of team composition, working relationships and therapeutic relationships. The topic guide consisted of three general domains: (1) the respondent’s role in the organisation, (2) collaboration between care providers within the same team and (3) collaboration with regard to care providers from other teams and departments. The interviews all started with the same opening questions per domain (‘What is your role within the organisation?’, ‘Who do you form a team with?’, ‘What does the collaboration with other teams and departments looks like?’). Further questions were then posed depending on the content of the interviewee’s answers in order to grasp their specific terminology and delve into the specifications of the work organisation, team composition, working relationships and therapeutic relationships.

Within each hospital, interviews were concentrated in time to ascertain that no changes in work organisation would occur during the period of investigation. We first contacted a hospital manager to inform him or her about the research. Next, a list of target key informants, that is, representatives from the medical, paramedical and nursing care providers involved in MS care, was composed by the first author on the basis of publicly available information on the hospital websites and through an on-site liaison, who also provided the necessary contact information. Interview times and locations were then directly scheduled with the key informants. Interviewees gave informed consent before the start of the interview and were able to stop the interview at any given time, but did not use this possibility. Interviews lasted between forty and ninety minutes, were conducted by the first author and a Master’s student, and were audio recorded. The recordings were privately and anonymously stored by the first author. Strict confidentiality and anonymity were ensured to the hospitals as well as to the interviewees.

To protect anonymity of the interviewees and the MS hospitals, we can only give the following, generalised information. The hospital number also does not correspond with the numbering in the Results for that reason. In total, 6 medical care providers, 11 paramedical care providers and 14 nursing care providers were interviewed, but the number of interviews per hospital differed. In the first hospital we examined, the number of interviews was higher (n = 18) because the data collection and analysis methods were being tested, ascertaining the number of interviews needed to correctly categorise the work organisation. In the first hospital, interviews were held with 2 medical care providers, 6 paramedical care providers and 10 nursing care providers. It soon became clear however that far less interviews were needed. In the second hospital, 6 interviews were conducted. The interviewees were 2 medical care providers, 2 paramedical care providers and 2 nursing care providers. In the third hospital, the interviewees were 1 medical care provider, 2 paramedical care providers and 1 nursing care provider (n = 4). In the fourth hospital, 1 care provider of each discipline (medical, paramedical, nursing) was interviewed (n = 3). Ultimately, interviews with one representative of each discipline proved to yield sufficient information for the data analysis.

### Data Analysis

We used the theoretical framework introduced earlier to frame our analysis and investigate the actual types of work organisation within the MS hospitals. The analytical procedure is intended to identify which type of work organisation was applicable to each of the cases [41]. Even though the hospitals’ work organisations were not explicated before the interviews, they soon surfaced in the topic interviews through instantaneous interpretation and follow-up questions. In a way, the process of data collection and analysis thus intersected, but this is not problematic because of two reasons. First, the a priori theoretical knowledge about possible work organisation differences guided the interviews as well as the concurrent interpretation of information and categorisation of the cases. Key informants’ comments regarding the way tasks were divided and grouped into organisational units were matched with the applicable work organisation, team composition, working relationships and therapeutic relationships type, with explicit attention for possible deviations from the archetypal conceptual definitions. Second, inter-researcher triangulation was organised to avoid subjectivity. The categorisation by the first author was thoroughly discussed to avoid mistakes and misinterpretations.

Ultimately, the four MS hospitals needed to be characterised by contrasting types of work organisation in order to be able to answer our research question. Each case was characterised by a different type of work organisation, so considerable variety was in fact found.

## Results

At first sight, the four MS hospitals appeared to be identical with regard to work organisation on the basis of their functional organograms. Not surprisingly, however, given the fact that organograms are notoriously flawed representations of what actually happens within an organisation [42], the work organisation was found to vary considerably in practice. While the first case did consist of an MS hospital that was characterised by a functional work organisation, the three other cases were characterised by more process-oriented types of work organisation. The following paragraphs render a detailed description of the work organisation per case, giving particular attention to the way care processes are shaped from the patient’s point of view, thus: from a systemic perspective. Table 1 summarises the findings with regard to the work organisation, team composition, working relationships and therapeutic relationships within the four MS hospitals.

**Table 1:** Work organisation, team composition, working relationships and therapeutic relationships in four MS hospitals.

Case	Work organisation	Team composition	Working relationships	Therapeutic relationships

1	Functional	Monodisciplinary	Ad hoc	Ad hoc
2	Process-oriented (partial)	Multidisciplinary	Ad hoc	Sequential
3	Process-oriented (partial)	Multidisciplinary	Fixed	Sequential
4	Process-oriented	Multidisciplinary	Fixed	Continuous

**Figure 1:**
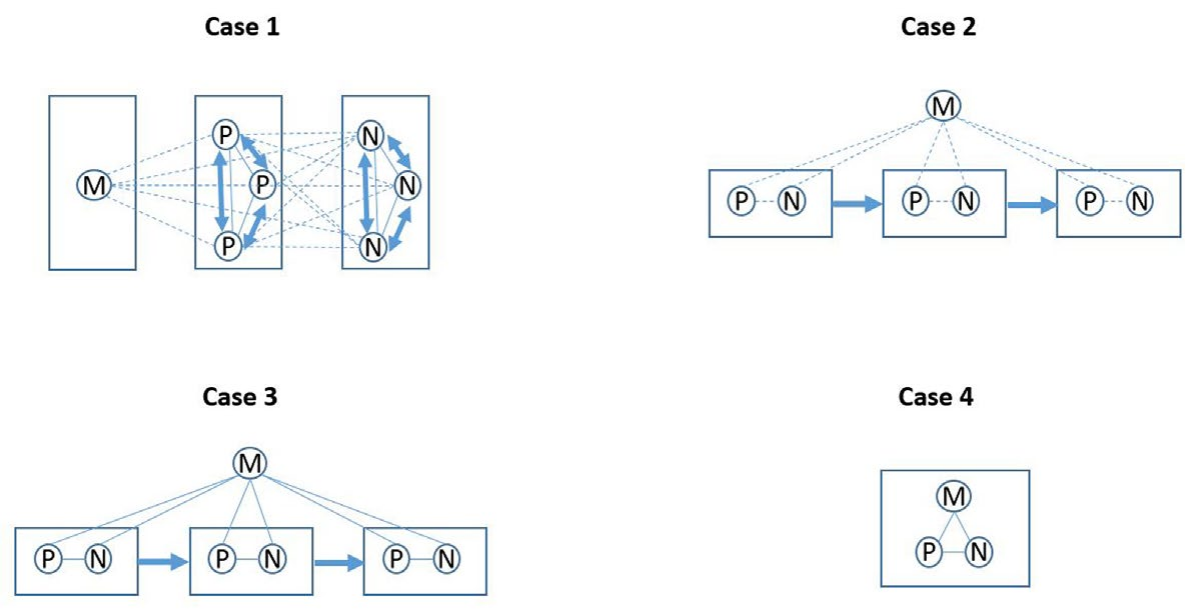
Work organisation, working relationships and therapeutic relationships in four MS hospitals. Figure 1 visualises the organisational units (squares), care providers (circles; Medical, Paramedical or Nursing), working relationships (lines; dotted = ad hoc, full = fixed) and therapeutic relationships (arrows; double = ad hoc, single = sequential, absent = continuous) per case.

Figure 1 visualises the organisational units (squares), care providers (circles), working relationships (lines) and therapeutic relationships (arrows) per case. The different care providers are symbolized by circles. M stands for medical care providers, P for paramedical care providers and N for nursing care providers. Dotted lines represent ad hoc working relationships, while the solid lines show fixed working relationships. The double arrows symbolize ad hoc therapeutic relationships, while the single arrows indicate sequential therapeutic relationships. The absence of an arrow means that there are continuous therapeutic relationships.

As already mentioned, the first case consisted of an MS hospital that was characterised by a functional work organisation. The composite task of delivering care for a patient was divided into distinct tasks which were grouped into organisational units per functional area. The monodisciplinary medical, paramedical and nursing departments and teams were in fact the organisational units to which tasks were allocated, contradicting the claim of multidisciplinary team presence within the hospital. Paramedical care providers did maintain fixed working relationships with the other paramedical care providers, and nursing care providers with other nursing care providers (solid lines in Figure 1), which renders teams monodisciplinary. Furthermore, all patients were followed-up upon by the same MD, but not by the same paramedical and nursing care providers. As one paramedical care provider pointed out:

“[The paramedical care provider] who is ready [to perform a particular task], goes inside and that means the patient is his for the time being. (…) we try to make sure that people don’t think they have an assigned therapist.”

Furthermore, medical, paramedical and nursing care providers did not cooperate on a fixed basis (dotted lines in Figure 1) as they were assigned ad hoc and individually to a particular patient without attention for continuity of care. Consequently, the patient’s care process was highly fragmented given that new therapeutic relationships had to be initiated constantly (double-headed arrows in Figure 1).

The second case consisted of an MS hospital that was characterised by a partial form of process-oriented work organisation. The composite task was divided into distinct tasks which were cross-functionally grouped into temporary multidisciplinary organisational units. In other words, an ad hoc multidisciplinary team was created each time a patient registered at the MS hospital, until the end of a non-predetermined care phase. As an interviewee explained:

“In principle, the team stays the same during one hospitalisation.”

This form of process-oriented work organisation was deemed partial because tasks were allocated to organisational units that related to a delineated phase of the care process instead of to the care process as a whole. The MD remained the same across care phases, thus throughout the care process as a whole, but paramedical and nursing care providers would be assigned ad hoc to serve a specific patient for the time of a particular care phase and, more importantly, they altered between the sequential care phases. This means that care providers did not maintain fixed working relationships with each other (dotted lines in Figure 1) and that the patient enters new therapeutic relationships at the start of each new care phase (single arrows in Figure 1). Again, the care process was fragmented, but to a lesser extent than in the first case.

The third case was also characterised by a partial form of process-oriented work organisation. The composite task was again divided into distinct tasks which were cross-functionally grouped into multidisciplinary organisational units per care phase. The difference with the second case is that fixed rather than ad hoc multidisciplinary teams were established linked to particular, predefined care programs. Care programs include short-term and long-term ambulatory rehabilitation, hospitalised rehabilitation, hospitalised medical intervention, and continuous hospitalisation. Consequently, care providers from different disciplines did work together on a regular, fixed basis in the context of the care program (solid lines in Figure 1). Yet, except for the MD, care was discontinuous in the transition between sequential care programs, when therapeutic relationships would be dismantled (single arrows in Figure 1). This specific work organisation constituted care fragmentation in the transition between different care programs.

The fourth case was characterised by process-oriented work organisation. The composite task was divided into distinct tasks which were cross-functionally grouped into multidisciplinary organisational units per care process. More specifically, MS patients would always be treated by the same multidisciplinary team of care providers. An interviewee commented:

“A couple of years ago we started working in clusters. We coupled [paramedical] therapists to [nursing] wards.”

Of course, some variability was possible due to, for instance, shift work, holiday arrangements and the general impossibility of 24/7 attendance. Nevertheless, care providers from different disciplines cooperated on a fixed basis (solid lines in Figure 1) and maintained continuous therapeutic relationships with the patient throughout the care process (absence of arrow in Figure 1). Consequently, the care process was integrated and continuous in this regard.

## Discussion

This article has two major objectives: providing a systemic view of multidisciplinary teams through the concept of work organisation and showing the associated implications by means of a comparative case study. In effect, the findings show the merits of a systemic perspective as it helps to clarify the similarities and differences that arise regarding team composition, working relationships and therapeutic relationships. The proclaimed presence of a multidisciplinary team in a care organisation is not necessarily reflected in practice. The claim might be incorrect as we saw in the first case, which consisted of an MS hospital where in fact monodisciplinary teams were in place. Moreover, the concept of multidisciplinary teams can be a black box hiding substantial differences in care processes. The second, third and fourth case did in fact consist of MS hospitals with multidisciplinary teams, but important variations were found. Multidisciplinary teams were limited to delivering care in a particular care phase (second and third case) or throughout the care process as a whole (fourth case), and working relationships were maintained on an ad hoc basis (second case) or on a fixed basis (third and fourth case). Work organisation differences were also reflected in the continuity of therapeutic relationships, which revealed whether the care process was fragmented or integrated.

The presented article extends on earlier research about work organisation differences, but such research is scarce in the healthcare literature as the topic is often overlooked. Studies on multidisciplinary teams use different definitions for the concept, but have in common that the broader work organisation is not referred to [1,2,43]. In addition, they mostly focus on topics such as professional cultures, communication and leadership [43,44]. West [45] famously studied healthcare team effectiveness and discerns between pseudo teams and real teams, which are respectively related to a functional and to a process-oriented work organisation. As our study shows, however, a lot more work organisation variation is to be found which explains differences in team composition as well as continuity of working and therapeutic relationships, and is expected to also have effects on health outcomes. Studies in other sectors show the relation between functional work organisations and negative outcomes with regard to organisational performance and quality of working life as well as the relation between process-oriented work organisations and positive outcomes in that regard, with the side note that functional work organisations do appear to be more productive in standardized mass production settings [46,47,48]. The originality and added value of our research lies in the systemic view that is advanced which shows the connections between often separately studied concepts: team composition, the continuity of working relationships, and the continuity of therapeutic relationships. Our findings are in line with what could be expected on the basis of theory as well as with the partial results of earlier research and research in other sectors [23,33,34,35,49]. This study thus helps to understand the potential connections between the findings from previous studies and can guide future research to be more integrated in this regard.

Taken together, the findings effectively show that the mere identification of a multidisciplinary team in a care integration study should be approached with caution if the team composition is not explicated in terms of work organisation throughout the care process as a whole, from the patient perspective. The current, fragmented understanding of these concepts [6] risks to overlook their interrelationships, which can only be understood through an in-depth, systemic understanding as advocated in our study. An encompassing systemic perspective on the way care-related activities are organised clarifies the role of different care integration components such as (supposedly) multidisciplinary teams. The concept of work organisation is key to this systemic perspective because it addresses the ‘core’ of a care system, namely the way care-related activities are organised, and allows to illuminate differences in this regard. Care integration taxonomies, models and typologies on care integration might find it useful to incorporate this kind of systemic thinking in order to establish a uniform understanding of multidisciplinary teams in practice. Care integration studies are expected to benefit from such thinking as it allows for realistic evaluation and, subsequently, a better informed comparison of care integration sites.

Albeit not being the focus of this article, the question is interesting why the hospitals organised their work organisation as they did. Some indications were observed for some of the hospitals. The second, partially process-oriented hospital seemed to have an underlying management philosophy. A care provider recounted that multidisciplinary teams were assembled ad hoc to avoid that patients would have the feeling that they have personally assigned care providers. No clear answer was given in this interview to the follow-up question as to why that feeling should be avoided, but a care provider in another hospital did say that patients can become overly exacting in a continuous therapeutic relationship. The third, partially process-oriented hospital also seemed to have an at least partially conscious management philosophy underlying the work organisation. Multidisciplinary teams were namely founded per care program, and those care programs were predefined reimbursement schemes by the government. The work organisation was thus modelled on the basis of these predefined care programs, even though the programs were not intended as such and other work organisation options were available as shown by the other hospitals. In the fourth, fully process-oriented hospital, a care provider declared that the goal of having one particular multidisciplinary team as a whole assigned to a patient population is that all care providers, disregarding their disciplinary background, know each other and each other’s patients. As already mentioned, work organisations often emerge within organisations rather than being designed consciously in most cases [36]. With regard to the hospitals investigated here, the question remains whether the underlying management philosophy was designed consciously and preceded the work organisation, whether the philosophy was conceived in hindsight to justify the work organisation, or if philosophy and work organisation emerged through a more complex, iterative, path-dependent process which combines the previous options of foresight and hindsight.

Future research could consider other study designs, even though the strengths of this study should be preserved. The comparative case study design namely brings along a number of limitations. Construct validity, researcher subjectivity and external validity are potential problems that need to be dealt with. Construct validity is the degree to which a measure actually gauges what it purports to be measuring. In the context of this study, the ‘measurement’ took place during the interviews as the first author interpreted interviewee statements, which pertains to the issue of subjectivity as well. The inter-researcher triangulation in this study increased the inter-rater reliability and therefore diminished the threat of researcher subjectivity and increased construct validity. Furthermore, external validity refers to the degree of generalisability of the results. Generally, a comparative case study encompasses a limited number of cases and therefore the results are not generalizable to the general population of cases. Nevertheless, the strategic selection of cases allows researchers to generalise results to a particular subset of the population. In the context of this study, the selection of the MS hospitals as cases allows us to generalise the results to care organisations that offer ambulatory and hospitalisation services to patient populations with severe chronic conditions. For that reason, the results need to be replicated in inter-organisational settings and for patient populations other than those with a severe chronic illness in order to explore the systemic consequences of work organisation in those contexts. Furthermore, we believe that data collection through topic interviews remains necessary for an in-depth understanding of cases, but at the same time we also see opportunities to maintain a limited number of work organisation categories, the measurement of process and outcome indicators and the execution of statistical analyses accordingly. The number of work organisation variations is potentially infinite, but a limited number of generalised categories can probably be defined, enabling statistical analysis. Importantly, process and outcome indicators should include measurements of patient experience and health outcomes, whose absence is an important limitation of the current article. Although we conceptualised the care process from the patient perspective, patient voice should also be incorporated more actively through assessments of and interviews on patients’ experiences and health outcomes. In sum, future research might want to consider a mixed-methods approach that preserves the strengths of the present research design in the context of a large N study with additional process and outcome measurements.

As mentioned in the introduction, a care system is also shaped by preconditions like legal and financial regulations as well as by culture, technology, education and other organisational aspects such as governance. Future research should also look into the relation with these topics. The CORTEXS research project [15] and, for instance, the COMIC model [50] try to take this into consideration. At the same time, we argue that a certain degree of conceptual parsimony might be useful to prioritise the fundamental understanding of what actually happens in a care system, before turning to the more extensive question ‘what works for whom under what circumstances?’ as posed by realistic evaluation.

Policymakers, managers, care providers as well as patients could also benefit from taking a systemic perspective, analysing the work organisation and reconfiguring multidisciplinary teams in the desired direction. This article does not merely illustrate the opposite types of work organisation, but a wider range of work organisation variation and the related differences in terms of team composition, working and therapeutic relationships. All stakeholders should be aware of and take into account work organisation specifics and their systemic implications.

A process-oriented work organisation helps care organisations move towards a more integrated care. As the Discussion already showed, however, a process-oriented work organisation and continuous therapeutic relationships specifically was not celebrated by all care providers because it potentially leads to overly demanding patients. Additionally, the manager of one hospital told us that a process-oriented work organisation would require too much staff. Theoretically, the work organisation type does not depend on the number of staff, but on the way staff is allocated to work units [39,40]. Nevertheless, in line with the previously discussed literature, this article finds that functional work organisation can be related to a more fragmented care, while process-oriented work organisation can be linked to a more integrated care.

## Conclusion

Our study shows by means of a comparative case study that a systemic perspective through the concept of work organisation reveals the different organisational arrangements that can underlie supposedly multidisciplinary teams. The actual composition of multidisciplinary teams, and the related working and therapeutic relationships will vary depending on the type of underlying work organisation. To be precise, the more process-oriented the work organisation, the more working relationships and therapeutic appeared to be fixed and continuous. Further validation of this conclusion will be needed in other settings, but the systemic perspective already allows for better evaluation, comparison and optimisation of care integration sites.

## Author Information

Sam Pless is a PhD candidate at the Centre for Sociological Research, KU Leuven (Belgium). His research interests include organisational, network and ecosystem (re)design in health care.

Geert van Hootegem is director of the Research Institute for Work and Society and senior Full Professor of Sociology at the KU Leuven (Belgium). He teaches organisational design and change management. His research on teamwork, new sociotechnical systems design and quality of working life is widely published in academic and management journals.

Ezra Dessers is an Assistant Professor of Work and Organisation Sociology at the Centre for Sociological Research, KU Leuven (Belgium). His research interests include workplace innovation, organisation design, inter-organisational collaboration and information technology.
